# Trends in Antidepressant Use and Expenditure in Six Major Cities in China From 2013 to 2018

**DOI:** 10.3389/fpsyt.2020.00551

**Published:** 2020-07-16

**Authors:** Zhenwei Yu, Jianying Zhang, Ying Zheng, Lingyan Yu

**Affiliations:** ^1^ Department of Pharmacy, Sir Run Run Shaw Hospital, College of Medicine, Zhejiang University, Hangzhou, China; ^2^ Department of Outpatient Nursing, Sir Run Run Shaw Hospital, College of Medicine, Zhejiang University, Hangzhou, China; ^3^ Department of Pharmacy, The 903 Hospital of the Joint Logistic Support Force of the PLA, Hangzhou, China; ^4^ Department of Pharmacy, Second Affiliated Hospital, College of Medicine, Zhejiang University, Hangzhou, China

**Keywords:** prescriptions, cost, selective serotonin reuptake inhibitor, tricyclic antidepressant, flupentixol/melitracen, escitalopram

## Abstract

The objective of this study was to investigate the current status and trends in antidepressant use and expenditure in China from 2013 to 2018. The study had a retrospective design based on prescribing data on antidepressant drugs, which was sourced from the Hospital Prescription Analysis Cooperative Project. The trends in hospital visits and corresponding expenditure on antidepressant drugs were examined. Subgroup analyses were carried out by sex, age, and drug class. A total of 1,795,230 outpatient prescriptions were collected from 79 hospitals in six major cities in China. Hospital visits with antidepressant prescriptions rose significantly from 244,626 in 2013 to 348,718 in 2018, reflecting a 42.6% increase (*P* < 0.05). The antidepressant expenditure also rose, increasing from 48.0 million Chinese yuan in 2013 to 64.8 million Chinese yuan in 2018. There were approximately 1.6 times more antidepressant prescriptions written for women than for men. The most frequent age category for antidepressant prescriptions was 45–64 years. The most commonly prescribed antidepressants were selective serotonin reuptake inhibitors (N06AB) and other antidepressants (N06AX), whereas tricyclic antidepressants (N06AA) accounted for only a small part of the total antidepressant prescriptions. Flupentixol/melitracen and escitalopram were the most frequently prescribed antidepressants. Antidepressant prescribing rates continue to increase in China, although the prescribing patterns have changed over the past few years. The wide use of expensive antidepressants and those with weak clinical evidence raises concerns regarding the rational use of antidepressants. This study provides a basis for future stewardship by the government and medical institutions.

## Introduction

Antidepressant medications are currently prescribed for a wide range of conditions, and an increasing trend in antidepressant prescriptions have been reported in many countries, including the United States, Australia, and some European countries (1–5). There are a number of reasons for this increase, including growing public awareness of depression, changes in patients’ attitudes, the expansion of therapeutic indications, and the impact of commercial interests (6, 7). In clinical practice, antidepressants are prescribed for many conditions other than depression, such as anxiety, sleeping disorders, psychosis, and neuropathic pain (3, 8). This has led to concern that antidepressants are being overprescribed.

The antidepressants available on the market have increased in recent years, providing prescribers with a variety of options. The choice of an antidepressant is influenced by drug profiles, physician characteristics, patient characteristics, regulation and reimbursement policies, and other factors (9). Antidepressants can induce several severe adverse effects, including self-inflicted injury, myocardial infarction, and stroke (10–12). Discontinuing or decreasing use of antidepressants, especially selective serotonin reuptake inhibitors (SSRIs), may lead to the appearance of withdrawal symptoms (13–15). Furthermore, concerns have been raised regarding whether the prescription of antidepressants is always justified (3). It is important to understand national trends and patterns of antidepressant use.

With the exception of one study that was restricted to patients with schizophrenia, little is known about the profile of antidepressant use in China (16). Therefore, this study aimed to describe the time trends and patterns of antidepressant prescriptions for adults from 2013 to 2018, using a large dataset.

## Methods

### Ethics

This study was approved by the Ethics Committee of Sir Run Run Shaw Hospital, College of Medicine, Zhejiang University (Reference Number: 20191011-18). Informed consent was waived as part of the approval.

### Study Setting and Population

This study was designed as a retrospective research based on prescription data. Antidepressant prescription data were obtained from the database of the Hospital Prescription Analysis Cooperative Project, which has been widely used in Chinese pharmacoepidemiology studies (17, 18). The database contained prescription information on sampling days of participating hospitals. There were 40 randomized sampling days per year, with 10 sampling days each quarter. For each prescription, the collected information included the prescription code, sex and age of the patient, prescription date, patient’s diagnosis, generic drug name, dose, and cost.

In the current study, outpatient prescriptions meeting the following criteria were extracted and included: (1) The prescriptions contained at least one antidepressant; (2) were prescribed for patients aged over 18 years; (3) were issued from 2013 to 2018; and (4) were issued in hospitals that participated in the program continuously during the study period and were located in Beijing, Tianjin, Shanghai, Guangzhou, Chengdu, or Hangzhou. No restrictions were imposed regarding indications because antidepressants are used to treat many conditions. However, prescriptions with incomplete information were excluded.

### Drug Classes

Antidepressant drugs were classified into five categories according to the World Health Organization Anatomical Therapeutic Chemical Index: (1) non-selective monoamine reuptake inhibitors (tricyclic antidepressants, TCAs): amitriptyline, clomipramine, doxepin, maprotiline; (2) N06AB, SSRIs: citalopram, escitalopram, fluoxetine, fluvoxamine, paroxetine, and sertraline; (3) N06AF, non-selective monoamine oxidase inhibitor; (4) N06AG, selective monoamine oxidase A inhibitor; and (5) N06AX, other antidepressants: agomelatine, bupropion, flupentixol/melitracen, reboxetine, neurostan, mirtazapine, mianserin, trazodone, duloxetine, milnacipran, and venlafaxine (4, 19).

### Data Analysis

The main units of analysis in this study were hospital visits where antidepressants were prescribed and expenditure on antidepressants. The number of visits was defined as the number of prescriptions meeting the inclusion criteria. Expenditure was defined as the cost of antidepressants. The yearly number of visits and expenditure were calculated, and the trends were analyzed.

Subgroup analyses by age, sex, and drug class were conducted. Three age groups were created to determine whether the trends in antidepressant use were being driven by a particular age group. The three age groups were young adults (18–44 years), middle-aged adults (45–64 years), and older adults (65 years and older).

The data were processed using Microsoft Access software. The rank-sum test was used to assess the statistical significance of trends for visits and expenditure. The Cochran–Armitage trend test was applied to determine the statistical significance of prescribing trends in drugs and drug classes. R V.3.3.0 (http://www.R-project.org) software was used for the statistical analysis.

## Results

### Total Trends in Antidepressant Visits and Expenditure

**Figure 1:**
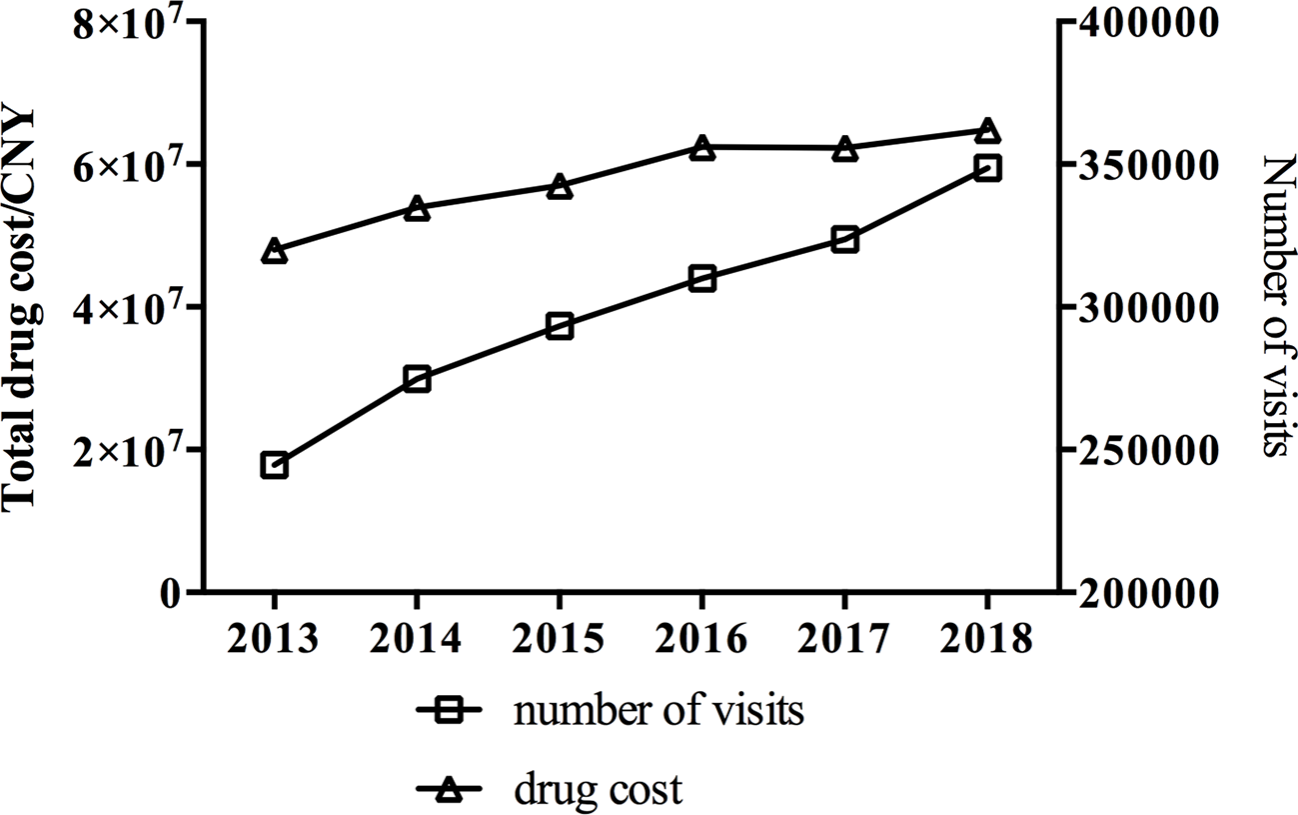
Trends in visits and cost of antidepressants in 79 hospitals located in six major cities in China from 2013 to 2018.

A total of 1,795,230 outpatient antidepressant prescriptions issued from 2013 to 2018 were reviewed in this study. These prescriptions were from 79 hospitals located in six major cities in China. All included hospitals were state-owned general hospitals. Of all the prescriptions, 42.1% were prescribed by psychiatrists and 33.0% were prescribed by neurological physicians. As **Figure 1** indicates, both visits and expenditure increased over time (both *P* < 0.05). The prescribing of antidepressant medications increased substantially from 244,626 in 2013 to 348,717 in 2018, reflecting a 42.6% increase over the study period. A 34.9% increase in prescribing costs was found over the same period—from 48 million Chinese yuan in 2013 to 64.8 million Chinese yuan in 2018.

### Trends by Age and Sex

We stratified the analysis of the trends in the number of antidepressant visits for any indication by age group and sex, and the results are shown in **Table 1**. The number of antidepressant visits for patients aged 18–44 years, 45–64 years, and 65 years and older all increased. Patients aged 45–64 years accounted for more than 40% of the visits. However, the percentage of older adult patients showed a small but significant increase over time (*P* < 0.05). Antidepressant drugs were more frequently prescribed to women, who accounted for 1.6 times more antidepressant visits than men (*P* < 0.05). However, the ratio of female-to-male users remained constant over the study period (*P* > 0.05).

**Table 1:** Demographic characteristics of included patients, 2013-2018.

	Number of patients (%)												
	2013		2014		2015		2016		2017		2018		
Age (years)													
18-44	69382	(28.36)	76073	(27.69)	80713	(27.52)	84434	(27.23)	88209	(27.25)	95589		(27.41)
45-64	107308	(43.87)	122116	(44.45)	129030	(43.99)	134851	(43.49)	139719	(43.16)	147865		(42.40)
65 up	67936	(27.77)	76550	(27.86)	83588	(28.50)	90792	(29.28)	95812	(29.60)	105263		(30.19)
Sex													
Male	90616	(37.04)	101609	(36.98)	109247	(37.24)	116328	(37.52)	119893	(37.03)	127958		(36.69)
Female	154010	(62.96)	173130	(63.02)	184084	(62.76)	193749	(62.48)	203847	(62.97)	220759		(63.31)

### Trends by Type of Drug

A total of 21 antidepressants in three drug classes were involved in this study. No instances of prescriptions for non-selective monoamine oxidase inhibitor (N06AF) or selective monoamine oxidase A inhibitor (N06AG) were observed. The antidepressant prescription visits and the percentage of the total prescriptions made up by each drug and drug class were examined, and the results are shown in **Table 2**. The total expenditure on antidepressant medications and the percentage of this accounted for by each drug are summarized in **Table 3**.

**Table 2:** Antidepressant prescription visits by drug and drug class, 2013-2018.

ATC code	Medicine	Number of visits (%)					
		2013	2014	2015	2016	2017	2018
N06AA		14827(6.06)	15634(5.69)	14834(5.06)	14606(4.71)	13373(4.13)	12790(3.67)
	Amitriptyline	8694(3.55)	9184(3.34)	8891(3.03)	8906(2.87)	8658(2.67)	8402(2.41)
	Clomipramine	731(0.30)	692(0.25)	655(0.22)	641(0.21)	654(0.20)	647(0.19)
	Doxepin	5155(2.11)	5533(2.01)	5084(1.73)	4867(1.57)	3877(1.20)	3554(1.02)
	Maprotiline	247(0.10)	225(0.08)	204(0.07)	192(0.06)	184(0.06)	187(0.05)
N06AB		126478(51.7)	134902(49.1)	145625(49.6)	156543(50.5)	161658(49.9)	172718(49.5)
	Citalopram	13637(5.57)	15727(5.72)	17707(6.04)	17024(5.49)	16216(5.01)	14207(4.07)
	Escitalopram	19850(8.11)	28585(10.4)	36162(12.3)	42182(13.6)	48379(14.9)	55279(15.9)
	Fluoxetine	13509(5.52)	14731(5.36)	12908(4.40)	12878(4.15)	11780(3.64)	11942(3.42)
	Fluvoxamine	4771(1.95)	6701(2.44)	7829(2.67)	8275(2.67)	8571(2.65)	9849(2.82)
	Paroxetine	42861(17.5)	37254(13.6)	36880(12.6)	39487(12.7)	39531(12.2)	42447(12.2)
	Sertraline	31850(13.0)	31904(11.6)	34139(11.6)	36697(11.8)	37181(11.5)	38994(11.2)
N06AX		103321(42.2)	124203(45.2)	132872(45.3)	138928(44.8)	148709(45.9)	163209(46.8)
	Agomelatine	133(0.05)	97(0.04)	49(0.02)	3(0.00)	293(0.09)	2255(0.65)
	Bupropion	482(0.20)	518(0.19)	575(0.20)	723(0.23)	951(0.29)	936(0.27)
	Duloxetine	10503(4.29)	14255(5.19)	14302(4.88)	17449(5.63)	20420(6.31)	23937(6.86)
	Flupentixol/melitracen	48685(19.9)	58622(21.3)	63769(21.7)	60977(19.7)	63824(19.7)	65617(18.8)
	Reboxetine	13(0.01)	9(0.00)	0(0.00)	0(0.00)	0(0.00)	0(0.00)
	Mianserin	623(0.25)	598(0.22)	766(0.26)	916(0.30)	1093(0.34)	1170(0.34)
	Mirtazapine	15482(6.33)	17780(6.47)	17621(6.01)	18592(6.00)	19660(6.07)	21489(6.16)
	Milnacipran	0(0.00)	0(0.00)	0(0.00)	8(0.00)	135(0.04)	210(0.06)
	Neurostan	352(0.14)	347(0.13)	83(0.03)	101(0.03)	535(0.17)	559(0.16)
	Trazodone	8552(3.50)	9977(3.63)	10869(3.71)	11950(3.85)	13595(4.20)	16295(4.67)
	Venlafaxine	18496(7.56)	22000(8.01)	24838(8.47)	28209(9.10)	28203(8.71)	30741(8.82)

**Table 3:** Total expenditure on antidepressant medications dispensed from 2013 to 2018 by drug and drug class.

ATC code	Medicine	Cost of Chinese yuan (%)					
		2013	2014	2015	2016	2017	2018
N06AX		162184(0.34)	150912(0.28)	139197(0.24)	134399(0.22)	115441(0.19)	144083(0.22)
	Amitriptyline	81596(0.17)	79001(0.15)	75502(0.13)	72435(0.12)	52052(0.08)	63106(0.10)
	Clomipramine	39563(0.08)	33029(0.06)	27268(0.05)	24916(0.04)	19019(0.03)	20180(0.03)
	Doxepin	28889(0.06)	28691(0.05)	26473(0.05)	27750(0.04)	35418(0.06)	52145(0.08)
	Maprotiline	12137(0.03)	10191(0.02)	9954(0.02)	9298(0.01)	8952(0.01)	8653(0.01)
N06AB		30366454(63.2)	31897367(59.2)	34035848(59.7)	37415695(60.0)	37273217(59.9)	38231262(59.0)
	Citalopram	3304822(6.88)	3918401(7.27)	4367593(7.66)	4172154(6.69)	3801416(6.11)	3069586(4.74)
	Escitalopram	6023948(12.5)	8369067(15.5)	10747229(18.9)	13046681(20.9)	14256349(22.9)	15536616(24.0)
	Fluoxetine	3659219(7.62)	3946896(7.32)	3272764(5.74)	3477740(5.57)	3042443(4.89)	2957276(4.56)
	Fluvoxamine	794085(1.65)	1103158(2.05)	1290382(2.26)	1433769(2.30)	1437000(2.31)	1560367(2.41)
	Paroxetine	10530022(21.9)	8498899(15.8)	7881856(13.8)	8256006(13.2)	7553850(12.1)	7691849(11.9)
	Sertraline	6054358(12.6)	6060945(11.2)	6476025(11.4)	7029344(11.3)	7182157(11.5)	7415567(11.4)
N06AX		17494875(36.4)	21874231(40.6)	22823643(40.0)	24842641(39.8)	24864451(39.9)	26414705(40.8)
	Agomelatine	72644(0.15)	60197(0.11)	37379(0.07)	1781(0.00)	124678(0.20)	687905(1.06)
	Bupropion	123598(0.26)	143183(0.27)	161264(0.28)	198178(0.32)	244859(0.39)	184247(0.28)
	Duloxetine	4042906(8.42)	5651351(10.5)	5402316(9.5)	6508445(10.4)	7025245(11.3)	7662359(11.8)
	Flupentixol/Melitracen	4221026(8.79)	5385611(9.99)	5855594(10.3)	5510436(8.8)	5140978(8.3)	4971748(7.7)
	Reboxetine	1230(0.00)	643(0.00)	0(0.00)	0(0.00)	0(0.00)	0(0.00)
	Mianserin	100041(0.21)	89928(0.17)	112878(0.20)	132846(0.21)	155082(0.25)	158740(0.25)
	Milnacipran	0(0.00)	0(0.00)	0(0.00)	3911(0.01)	51236(0.08)	74276(0.11)
	Mirtazapine	3070120(6.39)	3457236(6.41)	3320667(5.83)	3367417(5.40)	3273662(5.26)	3361283(5.19)
	Neurostan	46357(0.10)	50620(0.09)	11840(0.02)	15783(0.03)	83083(0.13)	91459(0.14)
	Trazodone	714002(1.49)	860164(1.60)	971800(1.70)	1068978(1.71)	1107519(1.78)	1307992(2.02)
	Venlafaxine	5102949(10.6)	6175297(11.5)	6949906(12.2)	8034867(12.9)	7658109(12.3)	7914696(12.2)

The most frequently prescribed antidepressant class was SSRIs, which remained in the leading position in terms of both the percentage of the total antidepressant prescription visits and the percentage of the expenditure during the study period. Although TCAs accounted for a small amount of both visits and costs, the percentages nevertheless decreased from 6.1% to 3.7% of antidepressant prescription visits and from 0.34% to 0.22% of the total cost from 2013 to 2018 (both *P* < 0.05). For the other antidepressant class (N06AX), a slight but continuous increase was seen in the percentage of all antidepressant prescription visits, which rose from 42.2% in 2013 to 46.8% in 2018 (*P* < 0.05). Over the same period, the expenditure on N06AX antidepressants increased from 36.4% to 40.8% of the total expenditure on antidepressants (*P* < 0.05).

The most frequently prescribed antidepressant was flupentixol/melitracen, followed by escitalopram, paroxetine, and sertraline. The visits where each of these four antidepressants was prescribed increased during the study period; however, escitalopram was the only antidepressant that showed an increase in terms of the percentage of antidepressant prescription visits where this drug was prescribed (all *P* < 0.05). The greatest antidepressant expenditure at the end of the study period was for escitalopram, followed by venlafaxine, paroxetine, duloxetine, and sertraline. The percentage of the total antidepressant expenditure accounted for by escitalopram increased dramatically from 12.5% in 2013 to 24.0% in 2018 (*P* < 0.05). In contrast, the percentage of the total expenditure accounted for paroxetine decreased from 21.9% in 2013 to 11.9% in 2018 (*P* < 0.05). Other antidepressants exhibited small and insignificant fluctuations.

## Discussion

This study examined the trends in antidepressant use in China using a large anonymized database. Progressive increases from 2013 to 2018 in the number of hospital visits where antidepressants were prescribed and in the expenditure on antidepressants were revealed in the study. We also analyzed antidepressants by age, sex, and drug class.

The overall trend in antidepressant use was similar to the trends that have been observed in other countries (20–23). This may suggest an increase in the burden of depression, the diagnosis of depression, or the prescription of antidepressants for conditions other than depression. Less than half of the antidepressant prescriptions examined in this study were issued by a psychiatrist, indicating that special concern should be paid to the rational use of antidepressants, particularly for indications other than depression. Antidepressant prescription visits increased among all age groups over the study period, whereas antidepressant prescription is generally found to be more frequent for patients aged 45–64 years. The antidepressant prescription visits of patients aged over 65 years increased relatively rapidly, and the percentage of visits accounted for by patients in this age group also increased. More attention should be paid to older adult users of antidepressants. In this study, we found that approximately 1.6 times more antidepressant prescriptions were prescribed to women than to men. This is not surprising, given that the prevalence and incidence of depression, as well as other psychiatric disorders such as anxiety and bipolar disorder, have previously been found to be higher for women than for men (20, 24–26). There was no difference in the sex ratio for antidepressant visits across the study years, which indicates that the relative distribution of the burden of depression and related conditions between men and women has not changed.

Our analysis showed that SSRIs accounted for nearly half of the antidepressant visits in China during the study period. However, our findings for the use of SSRIs were nevertheless lower than previous reports of SSRI use in other countries. In many countries, SSRIs are the predominant antidepressants, making up 60%–70% of the market (2, 27, 28). The clinical guidelines for treating depression suggest SSRIs (mostly escitalopram, paroxetine, and sertraline) as the preferred pharmacological treatment in adults (29, 30); however, the antidepressant efficacy of SSRIs is not superior to that of TCAs (31). SSRIs are also used to treat anxiety disorders, but the efficacy of SSRIs in this application is not superior to that of benzodiazepines (32). The reported overall adverse event rates of SSRIs and TCAs are comparable, but SSRIs are reported to have a lower incidence rate of cardiovascular effects and higher rates of neurogenic adverse effects (33). The percentage of the total antidepressant expenditure accounted for by SSRIs over the six years of study was almost 60%, indicating that SSRIs are relatively expensive. Considering that commercial interests have a major impact on the prescription of antidepressants, attention should be paid to the wide use of high-cost antidepressants (34, 35).

The prescription of TCAs was low despite these drugs having been the cornerstone of antidepressant pharmacotherapy for a long time. A similar trend has been observed in many other countries, although TCA use is still greater than SSRI use in Germany and an increasing trend has been reported for TCA use in the Netherlands (1, 4). The disadvantages of TCAs in terms of their cardiovascular side effects, high overdose fatality, and relatively great possibility of drug–drug interactions are responsible for the declining trend in TCA use (36, 37). However, other antidepressants—particularly SSRIs—also have serious problems because of withdrawal (14, 15). The higher use of TCAs than SSRIs in Germany suggests the important role of TCAs (1). The low price of TCAs is not a disadvantage, but this is another important reason for the decline (7).

A total of 21 antidepressant drugs were reported in this study. Flupentixol/melitracen, a mixture of a type of TCA and a classical antipsychotic component reported to be associated with significant improvement in quality of life independent of the presence of anxiety or depression, held the lead position in the antidepressant market from 2013 to 2018 (38). This is why the use of drugs in the other antidepressant class (N06AX) was much higher in our findings than in previous work in other countries. Some studies have suggested the efficacy of flupentixol/melitracen in treating resistant depression, ulcerative colitis, and several other conditions (39, 40). However, there is very limited evidence to support the use of this combination, and its popularity appears to coincide with marketing efforts. Flupentixol/melitracen is produced by a Danish company and is not registered in Denmark, the United States, the United Kingdom, Japan, or India (41). It can be assumed that flupentixol/melitracen is rarely used in these countries, despite the lack of reports on this. Special concern for safety and rational use should be raised regarding the wide use of this antidepressant with weak clinical evidence (42).

Escitalopram, the S-enantiomer of racemic citalopram, became the most frequently prescribed SSRI and moved into second place in terms of antidepressant prescriptions overall at the end of this study. This situation is quite different compared with other countries. The most commonly used SSRI in the United States, the United Kingdom, Italy, and Sweden has been shown to be citalopram, and sertraline has been reported as the most frequently used SSRI in Iran (2, 23, 24, 27, 43). Escitalopram is currently approved in over 100 countries and has been shown to have a good efficacy and safety profile (44–46). Escitalopram’s price varies by country. Although escitalopram was approved with a high price in some countries, previous studies have reported that escitalopram has a cost–utility advantage over other antidepressants in the treatment of major depression (47, 48). Escitalopram is expensive in China, elevating the total expenditure on SSRIs (**Table 3**). The pharmacoeconomic profile of the drug has not been evaluated for depression or other indications in China.

There are several limitations to this study. Our analysis was based on prescription data only; therefore, the appropriateness of the antidepressant therapy could not be evaluated, nor could the outcome of antidepressant therapy. Data on the indications for prescribing antidepressants were lacking, and this topic needs to be investigated in further studies. Finally, the prescription data were extracted from hospitals located in major cities in China, which might have induced bias.

## Conclusion

In this study, trends in antidepressant use and expenditure in six major cities in China were evaluated using a large database. Antidepressant prescription visits and expenditure on antidepressants were found to have increased over the study period. Furthermore, the most frequently prescribed antidepressants in this study differed from those reported in other countries. The wide use of antidepressants with weak clinical evidence or high costs raises concerns regarding the rational use of antidepressants. Using data from 79 hospitals in China from 2013 to 2018, this study provides a basis for future stewardship by the government and medical institutions.

## Data Availability Statement

All datasets generated for this study are included in the article/supplementary material.

## Ethics Statement

The studies involving human participants were reviewed and approved by Ethics Committee of Sir Run Run Shaw Hospital, College of Medicine, Zhejiang University. Written informed consent for participation was not required for this study in accordance with the national legislation and the institutional requirements.

## Author Contributions

Conceptualization: ZY, LY. Data curation: ZY, JZ. Formal analysis: LY, JZ, YZ, ZY. Funding acquisition: JZ, ZY. Investigation: LY, JZ, YZ. Methodology: ZY; Resources: ZY. Validation: LY, YZ. Visualization: LY. Writing-original draft: ZY, JZ. Writing—review and editing: YZ, LY.

## Funding

This work was funded by the Health Commission of Zhejiang Province and Traditional Chinese Medicine Administration of Zhejiang, China (2017KY089 and 2016ZQ027). The funders played no role in this research.

## Conflict of Interest

The authors declare that the research was conducted in the absence of any commercial or financial relationships that could be construed as a potential conflict of interest.
